# Catastrophic *Streptococcus pyogenes* Disease: A Personalized Approach Based on Phenotypes and Treatable Traits

**DOI:** 10.3390/antibiotics13020187

**Published:** 2024-02-15

**Authors:** Juan Carlos Ruiz-Rodríguez, Luis Chiscano-Camón, Carolina Maldonado, Adolf Ruiz-Sanmartin, Laura Martin, Ivan Bajaña, Juliana Bastidas, Rocio Lopez-Martinez, Clara Franco-Jarava, Juan José González-López, Vicent Ribas, Nieves Larrosa, Jordi Riera, Xavier Nuvials-Casals, Ricard Ferrer

**Affiliations:** 1 Intensive Care Department, Vall d’Hebron University Hospital, Vall d’Hebron Barcelona Hospital Campus, 08035 Barcelona, Spain; juancarlos.ruiz@vallhebron.cat (J.C.R.-R.); carolina.maldonado@vallhebron.cat (C.M.); adolfo.ruiz@vallhebron.cat (A.R.-S.); laura.martinsanchez@vallhebron.cat (L.M.); ivanrene.bajana@vallhebron.cat (I.B.); juliana.bastidas@vallhebron.cat (J.B.); jordi.rieradelbrio@vallhebron.cat (J.R.); xavier.nuvials@vallhebron.cat (X.N.-C.);; 2 Shock, Organ Dysfunction and Resuscitation Research Group, Vall d’Hebron Research Institute (VHIR), Vall d’Hebron University Hospital, Vall d’Hebron Barcelona Hospital Campus, 08035 Barcelona, Spain; 3 Departament of Medicine, Universitat Autonoma de Barcelona, 08193 Barcelona, Spain; 4 Immunology Department, Vall d’Hebron University Hospital, Vall d’Hebron Barcelona Hospital Campus, 08035 Barcelona, Spain; rocio.lopez@vallhebron.cat (R.L.-M.); clfranco.hj23.ics@gencat.cat (C.F.-J.); 5 Microbiology Department, Vall d’Hebron University Hospital, Vall d’Hebron Barcelona Hospital Campus, 08035 Barcelona, Spain; juanjo.gonzalez@vallhebron.cat (J.J.G.-L.); nieves.larrosa@vallhebron.cat (N.L.); 6 Eurecat, Centre Tecnològic de Catalunya, EHealth Unit, 08005 Barcelona, Spain; vicent.ribas@eurecat.org

**Keywords:** severe sepsis, septic shock, multiorgan dysfunction, precision medicine, hemoadsorption, *S. pyogenes*, sepsis phenotypes, streptococcal toxic shock syndrome

## Abstract

Streptococcal toxic shock syndrome (STTS) is a critical medical emergency marked by high morbidity and mortality, necessitating swift awareness, targeted treatment, and early source control due to its rapid symptom manifestation. This report focuses on a cohort of 13 patients admitted to Vall d’Hebron University Hospital Intensive Care Unit, Barcelona, from November 2022 to March 2023, exhibiting invasive *Streptococcus pyogenes* infections and meeting institutional sepsis code activation criteria. The primary infections were community-acquired pneumonia (61.5%) and skin/soft tissue infection (30.8%). All patients received prompt antibiotic treatment, with clinical source control through thoracic drainage (30.8%) or surgical means (23.1%). Organ support involved invasive mechanical ventilation, vasopressors, and continuous renal replacement therapy as per guidelines. Of note, 76.9% of patients experienced septic cardiomyopathy, and 53.8% required extracorporeal membrane oxygenation (ECMO). The study identified three distinct phenotypic profiles—hyperinflammatory, low perfusion, and hypogammaglobulinemic—which could guide personalized therapeutic approaches. STTS, with a mean SOFA score of 17 (5.7) and a 53.8% requiring ECMO, underscores the need for precision medicine-based rescue therapies and sepsis phenotype identification. Integrating these strategies with prompt antibiotics and efficient source control offers a potential avenue to mitigate organ failure, enhancing patient survival and recovery in the face of this severe clinical condition.

## 1. Introduction

A significant increase in invasive group A streptococcal (GAS) infections has been observed since September 2022 [1,2] in several European countries and the USA. In our geographical area different series have already been published, showing an increase in their incidence [3]. Streptococcal toxic shock syndrome (STSS) is a critical life-threatening condition, including invasive infections principally by group A streptococcus (GAS, *S. pyogenes*) [4].

### 1.1. Streptococcal Pathophysiology

Streptococcal infections are quite prevalent and can result in a range of conditions, varying from self-limiting to severe disease, including bacteremia, pneumonia, meningitis, endocarditis, arthritis, sinusitis, and infections of soft tissues such as necrotizing fasciitis and myositis [5]. The precise mechanism of STSS is not fully understood. However, it is linked to a cascade of effects involving streptococcal toxins, including enterotoxins with superantigenic properties and other streptococcal enzymes and toxins. These elements interact with the host immune system in a particular manner [6]. 

One commonly accepted premise is that bacterial pathogens must initially establish close contact with extracellular matrix proteins (ECM) in host cells to establish a successful infection. Highly specific adhesins facilitate this initial interaction with ECM proteins or cells. These adhesins and other macromolecules that provoke the internalization of bacteria or the invasion of host cells are collectively called “invasins”. Streptococci, in particular, is characterized by an extensive and highly variable array of adhesins and invaders. The expression of these proteins is subject to differential regulation and is influenced by signals from various environments within the human host [7,8]. Given the diverse repertoire of adhesins and invasins in streptococci, these pathogens have developed multiple strategies to achieve internalization and survival within host cells, including evading antibiotic actions and host immune responses [9,10].

Recognization and attachment to diverse ECM proteins are crucial steps in the streptococcal colonization of human tissues. Firstly, lipoteichoic acid (LTA) is thought to mediate the first step of adhesion with low cellular specificity [11]. Then, the expression of the hyaluronic acid (HyA) capsule protects against phagocytosis by immune system cells. The HyA capsule can act as a non-protein adhesin by binding to the HyA receptor CD44 on skin keratinocytes [12]. Most importantly, the interaction between HyA and CD44 receptors leads to cytoskeletal rearrangements in human epithelial cells. These rearrangements disrupt intracellular junctions and allow the dissemination of streptococci into deeper, underlying sterile tissue [13]. Models of human skin indicated that cell signaling triggered by the interaction of the GAS capsule with CD44 opened intercellular junctions and promoted tissue penetration by GAS through a paracellular route with no evident portal of entry on many occasions [14]. M protein [15] and the C5a peptidase [16] also contribute to skin invasion, granting bacterial aggregation, which may also be crucial for colonization, resistance to phagocytosis, and the subsequent invasion of cells. Next, in a stationary phase, once the invasion has occurred, the production of exotoxins, such as streptolysin, streptokinase, or hyaluronidase, triggers platelet and leukocyte aggregation, and, more importantly, the production of superantigens leading to the polyclonal activation of lymphocytes, the inflammatory cytokine storm cascade, and the state of shock, the third phase of GAS infection.

Classically, the clinical presentation of STSS is described in three phases. The third one, which is the one we will focus on, is characterized by a severe distributive shock associated with multiple organ dysfunction. Despite aggressive therapy, many patients will die within 24 to 48 h of admission.

### 1.2. Streptococcal Pathophysiology Related to Septic Shock Management

The latest edition of the Surviving Sepsis Campaign (SSC) guidelines underscores the necessity for precise and protocolized care in managing septic shock [17]. Implementing such measures is linked to a significant decrease in mortality [18]. However, mortality from infection in its sepsis version remains high [19]. Certain patient subsets unresponsive to standard treatments might find relief in alternative therapies, viewed as rescue interventions. The existing consensus description of sepsis, encompassing “life-threatening organ dysfunction resulting from a dysregulated host response to infection”, is expansive, capturing the inherent diversity of the ailment [20]. The diversity in this situation stems from multiple factors: varying infection causes, unique comorbidities and genetics in individual hosts, and differences in the timing of diagnosis and treatment among patients. These elements influence the progression of sepsis at the individual patient level and their reactions to therapeutic interventions. 

Seymour et al. [21], utilizing an extensive database, characterized four distinct sepsis phenotypes based on varied demographics, laboratory parameters, and patterns of organ dysfunction. These were found to correlate with biomarker concentrations and mortality rates. The identification of these phenotypes suggests the potential benefit of more targeted and personalized therapeutic approaches for patients at a heightened risk of clinical deterioration [22]. The emergence of these challenges underscores the importance of precision medicine. Precision medicine enables the customization of healthcare interventions for specific patient groups, taking into account factors such as disease susceptibility, diagnosis, prognosis, and treatment response.

### 1.3. Objectives of This Study

In this report, the objective is to describe a population of patients who are mostly in severe septic shock and multiorganic dysfunction due to STSS, highlighting certain aspects of the inflammatory and immunological response that can determine the application of precision medicine. 

## 2. Results

Our study includes a population of 13 patients (demographic and clinical characteristics are described in Table 1, and analytical characteristics during the first 24 h are described in Table 2) without any significant comorbidity. The predominant infection source was community-acquired pneumonia (61.5%) and skin and soft tissue infections (30.8%). Regarding microbiology, positive peripheral blood cultures were identified in five (38.5%) cases. Notably, there were seven viral coinfections mainly due to the Influenza B virus (FLUBV) (38.5%). No previous use of NSAIDs was reported.

### 2.1. General Management and Conventional Organ Support

All patients received early antibiotic treatment with Penicillin G and Clindamycin, in addition to empirical Meropenem, within the first 72 h of disease onset. Meropenem was withdrawn after other sources of infection were ruled out. Clindamycin [23] was used for 3 days in all cases, and Penicillin G was employed until resolution of the source of infection (extended regimens were administered in cases of necrotizing pneumonia or skin wounds until clinical or radiological resolution). Thus, individualized regimens were prescribed. Source control was performed by thoracic drainage (30.8%) or other measures for surgical control (23.1%). Organ support was provided through invasive mechanical ventilation (IMV) [24]. Vasopressors and continuous renal replacement therapy (CRRT) [25] were also administered in compliance with clinical guidelines. Corticosteroid therapy, in the form of hydrocortisone administered at 50 mg intervals every six hours, was incorporated as a component of septic shock management. Hydrocortisone has been used until withdrawal of vasoactive drugs or resolution of shock, with tapering if administered for more than seven days.

### 2.2. Personalized Treatment

Our study focused on a subgroup of patients with severe septic shock who met the criteria for STSS, as indicated by a mean SOFA score of 17 (with a standard deviation of 5.7) points. We describe different phenotypes that may result in a personalized approach based on treatable traits: hyperinflammatory, low perfusion, or hypogammaglobulinemic profile. 

Implementing this multi-faceted approach was based on phenotypic considerations; regardless of an anticipated high mortality risk predicted to exceed 50% according to SOFA or APACHE II criteria [26,27,28], the survival rate was 77%.

#### 2.2.1. Hyperinflammatory Phenotype

Personalized therapies were employed utilizing cytokine hemoadsorption (HA) (Cytosorb^®^ (CytoSorbents (Corporation, Monmouth Junction, NJ, USA)) and endotoxin HA (Toraymixin^®^ (Toray, Tokyo, Japan)) (76.9%), guided by biomarker assessment. Specifically, patients treated with Cytosorb^®^ or Toraymixin^®^ exhibited plasmatic concentrations of IL-6 of 108, 110.45 (104,325) pg/mL and Endotoxin Activity (EAA) of 0.93 (0.33), respectively. A sequential HA (cytokine HA followed by endotoxin HA) was implemented in six (46.2%) cases. 

#### 2.2.2. Low Perfusion Phenotype

Notably, 76.9% of cases exhibited septic cardiomyopathy coupled with severe multiorgan dysfunction. Moreover, 53.8% of patients were supported by extracorporeal membrane oxygenation (ECMO) therapy, either as a response to distributive shock (specifically, five (38.4%) patients required venoarterial ECMO] or in the face of refractory hypoxemia.

#### 2.2.3. Low Immunoglobulin Phenotype

Hypogammaglobulinemia was defined by gammaglobulins (IgG) levels falling below 500 mg/dL or two standard deviations below reference values for age [29]. The administration of 2 g/kg of polyclonal gammaglobulin was promptly initiated upon clinical suspicion of STSS. Furthermore, patients showing plasma IgG concentrations below 500 mg/dL received 250 mg/kg/d administered via a 10 h infusion over three consecutive days. Nine (69.2%) patients exhibited low immunoglobulin phenotype.

## 3. Discussion

Sepsis and septic shock should be considered diverse diseases encompassing various clinical trajectories and distinct phenotypic expressions. Given its association with heightened mortality risk, patients afflicted with this condition necessitate a meticulously structured and protocol-driven therapeutic approach. The fundamental sepsis management principles, including infection control, initial resuscitation, and comprehensive organ support, form the cornerstones of treatment. Yet, personalized interventions targeting specific pathophysiological mechanisms can significantly benefit specific patient phenotypes. In the context of STSS, combining immunoglobulin and hemoperfusion may benefit severe cases with septic shock and multiple organ failure admitted to the ICU [30,31], complementing the core management bundles. Within our studied population, we identified three distinct phenotypic profiles that merit discussion: the hyperinflammatory profile characterized by elevated cytokine levels or pronounced endotoxemia, the low perfusion phenotype, and the low immunoglobulin (Ig) phenotype (Table 3).

In the current body of literature, a range of terms have been used to describe presentations of septic shock, particularly those indicating a poor prognosis. In critically ill adult patients, commonly used terms include “refractory septic shock”, “catecholamine resistance”, or “high dose norepinephrine”. However, it is essential to highlight that there is no universally agreed-upon consensus regarding exact definitions for these medical situations [32]. Distinguishing between phenotypes of patients with severe septic shock due to STSS based on identifiable treatable features has the potential to improve patient outcomes.

### 3.1. Hyperinflammation with High Cytokine Phenotype

The use of HA therapy as a rescue therapy may be considered in a situation of septic shock and multiorgan dysfunction refractory to standard treatment. Cytokine HA may have a role as rescue therapy in a particular subgroup of patients with severe septic shock, hyperlactatemia, multiorgan failure, and very high hypercytokinemia. There is no threshold of plasma cytokine levels for initiating or withholding the therapy. In this context, blood purification techniques have the potential to attenuate the inflammatory process, exerting a swiftly substantial, non-selective impact on the cytokine storm. Using adsorption cartridges in STSS may introduce a novel therapeutic approach for severe cases. Their effectiveness in regulating proinflammatory mediators, which play a significant role in cellular toxicity and organ dysfunction, could prove pivotal in lowering the mortality rate [33]. Plasmapheresis has been proposed to manage STSS, but the evidence for its use is still low, based on isolated case reports [34]. Still, certain pharmacokinetic drawbacks of HA cannot be overlooked, such as variations in antibiotic plasmatic concentrations [35]. In any case, it is important to monitor antibiotic levels, specifically when the periods of HA are long. Cytokine HA is a safe procedure without relevant associated adverse effects [36]. In our case, no adverse effects were observed. 

### 3.2. Hyperinflammatory with High Endotoxemic Phenotype

Endotoxin has been identified as one of the therapeutic targets for sepsis and septic shock. The elimination of endotoxin through blood purification techniques, specifically via HA, has been proposed [37]. Endotoxemia and the excessive production of inflammatory mediators, manifested as a cytokine storm, are linked to the severity of sepsis and septic shock, influencing patient prognosis [38]. Klein et al. [39] performed a post-hoc analysis on the Euphrates trial [40] on 194 patients with EAA between 0.6–0.89. A survival benefit was observed in patients who received polymyxin (PMX) hemofilter therapy. In a review of recent studies, Shoji et al. [41] demonstrated a survival benefit of PMX hemoperfusion. Lately, in a multicenter, prospective, and observational study, it was reported that the baseline EAA may predict the outcome of critically ill septic patients receiving PMX-HA [42]. It is thus justifiable to consider patients experiencing septic shock and severe multiorgan dysfunction, with well-managed control of the infectious source and an EAA of 0.6–0.9, as potential candidates for endotoxin HA. 

### 3.3. Sequential Approach

Current practice has shown that HA helps in the recovery of immune homeostasis. However, for certain patients, endotoxin-only adsorption may be insufficient [43]. Sequential HA (endotoxin HA with PMX, Toraymixin^®^, and subsequent cytokine HA with Cytosorb^®^) has been implemented in highly selected patients [44]. Sequential HA is intended to remove the primary stimulus that induces the dysregulated inflammatory response. Candidates for sequential HA include patients experiencing septic shock, multiorgan dysfunction, elevated endotoxemia, and hypercytokinemia (particularly extremely high levels of IL-6). Continuous monitoring of plasma cytokines (IL-6, IL-10) can provide guidance to clinicians for therapy management. 

### 3.4. Low Perfusion Phenotype

We introduced the term “low perfusion” to categorize patients experiencing septic cardiomyopathy, characterized by insufficient perfusion even when on vasoactive agents and receiving supportive treatment tailored to other phenotypes. The use of mechanical circulatory support continues to be a subject of debate in the management of refractory septic shock among adult patients [45]. The venoarterial (VA) configuration of ECMO support presents an appealing choice for shock management, particularly in patients experiencing severe concurrent cardiac and pulmonary failure. However, high-quality evidence supporting its use in adults is still limited. Riera et al. [46] conducted a review highlighting extracorporeal membrane oxygenation (ECMO) as a supportive method rather than a treatment. The authors pointed out that with an appropriate configuration and well-defined management, this method may rescue specific adult septic patients with no other survival options.

### 3.5. Low Immunoglobulin Phenotype

The supplemental utilization of intravenous immunoglobulin (IVIG) in STSS is theoretically justified by its anti-inflammatory and immunomodulatory properties. Additionally, supporting evidence comes from observational studies and one historically controlled trial [47]. The INSTINCT trial found no significant distinction between the placebo and intervention groups in relation to the primary outcome, which involved evaluating functional status through the Physical Component Summary (PCS) score of the 36-item short-form health survey (SF-36) six months after randomization. In addition, mortality and multiple organ failure did not show differences. However, it is important to note that patients with confirmed STSS accounted for less than 10% of the study population, making it challenging to draw definitive conclusions for this subgroup. In line with guidelines from the Infectious Diseases Society of America (IDSA), there is an emphasis on the necessity for further studies to assess the efficacy of IVIG for this particular indication [48]. Hence, recommending the routine use of IVIG in STSS is not feasible, and decisions should be made case-by-case. SSC guidelines provide a weak recommendation for the use of immunoglobulins (Ig) as a treatment for septic patients due to the low certainty of evidence in the main studies [49] and meta-analyses [50]. This SSC recommendation should be limited only to patients with normoglobulinemia since the studies have not assessed the status of immunoglobulins as an inclusion criterion. However, it is reasonable that in septic shock and multiorgan failure, IVIG could be beneficial in the presence of hypogammaglobulinemia. Polyvalent intravenous immunoglobulins represent a promising approach to modulating pro- and anti-inflammatory responses [51]. The optimal dosing and timing of administration of IVIG in STSS is uncertain. The treatment with polyclonal gammaglobulin of 1 or 2 g/kg is accepted (level of evidence 2C) [52]. In another study, projecting information beyond the existing data, RCTs in Kawasaki disease were recommended for early treatment with a high dose (2 g/kg) [53]. Other strategies proposed IgM- and IgA-enriched polyclonal IVIG doses of 250 mg/kg/d using a 10 h infusion for 3 consecutive days [54]. 

### 3.6. STSS and Its Relation to Precision Medicine Guided by Phenotypes

There is considerable variability in sepsis features among patients due to differences in age, causative microorganisms, focus type, and patient comorbidities. At the pathophysiological level, the inflammatory response manifests in two stages: the proinflammatory response and the anti-inflammatory response, which can differ among individuals or even within the same individual based on the severity continuum they are experiencing.

The emergence of initiatives aimed at enhancing sepsis diagnosis and treatment has been observed in recent years. Precision medicine introduces a novel approach to patient management, allowing specific subgroups to benefit from therapeutic strategies that may not be suitable for the general sepsis patient population. Sepsis management involves well-structured, protocolized interventions subject to continuous revision, as emphasized throughout the manuscript. While all patients should undergo the fundamental pillars of sepsis treatment—infection control, initial resuscitation, and multiorgan support—certain subpopulations may benefit more from tailored therapies.

Due to the diverse nature of sepsis, clinical progression varies among patients. Low-risk patients may undergo conventional management to ensure a favorable prognosis. In contrast, high-risk patients, prone to organ dysfunction and mortality, may necessitate adjuvant-specific therapies aligned with their pathophysiological characteristics. This diversity underscores the emergence of precision medicine in sepsis, aiming to individualize the management of septic patients by identifying distinct endotypes or subgroups based on genetic or biological dissimilarities. Precision medicine facilitates the characterization of homogeneous patient subgroups exhibiting similar evolutionary patterns and responses to specific treatments.

Certainly, we acknowledge that treatments based on these phenotypes are not yet supported by evidence in the form of randomized clinical trials. Therefore, they are not recommended by the SSC guidelines. We want to emphasize this point conscientiously. However, subgroups of patients refractory to conventional treatments could benefit from specific treatments based on phenotype characterization. These are patients with a high risk of mortality and are often not included in randomized studies. Their accurate identification and selection could lead to an improvement in survival rates.

In our series, it is imperative to underscore the timely initiation of antibiotic therapy and source control and the integration of precision medicine tailored to sepsis phenotypes, incorporating gammaglobulin supplementation. The application of hemoadsorptive treatments, guided by real-time monitoring of cytokine levels and endotoxemia, along with the strategic utilization of ECMO in its various configurations, has culminated in a survival rate of 77%. This outcome notably surpasses the mortality rates anticipated by the SOFA and APACHE II scoring classification systems.

In the realm of precision medicine, focusing on STSS, interventions for modulation of the inflammatory cascade can include the use of antibodies to inhibit lymphocyte activation and subsequently mitigate the cytokine storm. For instance, Reltecimob, a CD28 T-lymphocyte receptor mimetic designed to suppress T-cell stimulation, has been developed. Bulger et al. [55] administered this treatment within six hours of diagnosing necrotizing soft tissue infections via surgical methods in patients with a SOFA score of ≥3. The study involved 290 patients, with an average screening SOFA score of 5.5 ± 2.4, and the results demonstrated improved resolution of organ dysfunction and time to hospital discharge.

In approaching refractory patients, a multidisciplinary approach is imperative, from the comprehensive perspective of the intensivist to the data evaluated by the immunologist and microbiologist to adjust the treatment in real-time.

Although our study included a small sample, implementing this multi-faceted approach was based on phenotypic considerations. Despite the anticipated high predicted mortality risk exceeding 50% according to SOFA or APACHE II criteria, the survival rate was 77%.

### 3.7. Limitations

This study has some limitations. First, this single-center study includes a small number of patients with no control group. Thus, the findings of these observations should be confirmed in larger comparative studies and cannot be extrapolated to other ICU settings. We described a very selected profile of patients in septic shock and multiorgan dysfunction with real-time monitoring of cytokines and high endotoxemia. Therefore, our conclusions should not be applied to all types of patients.

## 4. Materials and Methods

### 4.1. Study Design and Setting, Inclusion Criteria and Analyzed Data and Scores

The present retrospective study included all patients admitted to the Intensive Care Unit (ICU) of Vall d’Hebron University Hospital, Barcelona, Spain, with invasive infection by *Streptococcus pyogenes* during the period between November 2022 and March 2023. 

Patients met the criteria for activating the Vall d’Hebron University Hospital in-hospital sepsis code (ISC). The Centers for Disease Control and Prevention’s (CDC’s) definition of STSS was followed [30,56]. The data collected included demographic variables, past medical history and clinical and analytical variables. The severity of the disease was evaluated through the Acute Physiology and Chronic Health Disease Classification System (APACHE II) [57] and Sequential Organ Failure Assessment (SOFA) scores [58]. Both scores were calculated using the worst parameters measured during the first 24 h of ICU admission. The presence of septic shock or sepsis was assessed according to the Sepsis 3 criteria [20]. ARDS was assessed in accordance with the Berlin definition criteria [59]. Data on the incidence of acute kidney injury (AKI) or failure and the need for continuous renal replacement therapy (CRRT), were collected in line with the latest Kidney Disease: Improving Global Outcomes (KDIGO) Clinical Practice Guideline criteria [25]. Length of ICU stay and ICU and in-hospital mortality were also registered. 

Using clinical evaluation and biomarker determination, we identified different phenotypes that may result in a personalized approach based on treatable traits: hyperinflammatory profile, low perfusion profile, and hypogammaglobulinemic profile. Patients were treated accordingly as previously described.

### 4.2. Statistical Analysis

We used the Statistical Package for the Social Sciences SPSS Statistics 18.0 software (SPSS Inc., Chicago, IL, USA) for statistical analyses. Continuous variables were tested with the Kolmogorov–Smirnov test. Qualitative or categorical variables were described as numbers and percentages. Quantitative or continuous variables were described as means, standard deviations, medians, and interquartile ranges depending on their probabilistic distributions. 

### 4.3. Ethics Statement

The data collection of the study is supported by the local Clinical Research Ethics Committee (PR (AG) 336/2016). The study complied with the General Data Protection Regulation (GDPR) (Regulation (EU) 2016/679), and was performed in accordance with the ethical standards in the 1964 Declaration of Helsinki and its later amendments. The committee complies both in its composition and in the Standard Work Procedure (SWP) with the Best Clinical Practice (BCP) standards (CPMP/ICH/135/95) and with Royal Decree 1090/2015. The datasets used and analyzed during the current study are available from the corresponding author upon reasonable request.

## 5. Conclusions

Streptococcal Toxic Shock Syndrome (STSS) constitutes a profoundly severe clinical condition associated with a notably high mortality rate. Prospective therapeutic approaches that integrate targeted interventions rooted in precision medicine and discerning sepsis phenotypes, in conjunction with early administration of antibiotics and proficient source control, could improve patient outcomes by mitigating the rapid progression towards multiorgan failure. The multidisciplinary approach involving diverse medical disciplines plays a pivotal role in discerning distinctive characteristics, commonly referred to as phenotypes, in septic patients.

## Figures and Tables

**Table 1:** Characteristics of the study population. The data have been expressed as “n” (%) if they are categorical and as median (interquartile range) or mean (standard deviation) if they are quantitative.

		n = 13
**Age** [years, m (SD)]		42 (13.8)
**Gender** [n (%)]	Female	7 (53.8)
	Male	6 (46.2)
**Comorbidities** [n (%)]	Hypertension	3 (23.1)
	Obesity	1 (7.7)
	Cardiac	1 (7.7)
	Inmunosuppression	2 (15.4)
	Peripheral Vascular Disease	1 (7.7)
**APACHE II** [m (SD)]		29 (15)
**SOFA** [m (SD)]		17 ± 5.7
**Infection source** [n (%)]	Respiratory	8 (61.5)
	SSTIs	4 (30.8)
	PID	1 (7.7)
**Source control** [n (%)]	Thorax Drainage	4 (30.8)
	Surgery	3 (23.1)
**Coinfection** [n (%)]	FLUB-V	5 (38.5)
	Metapneumovirus	2 (15.4)
**Microbiologic culture-positive tests**	Isolated PBC [n (%)]	5 (38.5)
	PBC + BAL [n (%)]	2 (15.4)
	Bronchoalveolar lavage [n (%)]	4 (31)
	Skin/soft tissue [n (%)]	1 (7.7)
	PBC + Ascitic fluid [n (%)]	1 (7.7)
**Septic Shock** [n (%)]		13 (100)
**Septic Cardiomyopathy** [n (%)]		10 (76.9)
**Cardiovascular SOFA** [m (SD) ]		4 (0.6)
**Dobutamine use** [n (%)]		10 (76.9)
**Hydrocortisone** [n (%)]		13 (100)
**IMV** [n (%)]		9 (69.2)
**ECMO (VA. VV)** [n (%)]	Total [n(%)]	7 (53.8)
	VA [n(%)] Low perfusion phenotype	5 (38.4)
**CRRT** [n (%)]		10 (76.9)
**HA** [n (%)]	(total)	10 (76.9)
	Cytosorb^®^	9 (69.2)
	Toraymixin^®^	6 (46.2)
	Oxiris^®^	2 (15.4)
**Sequential hemoadsorption** [n (%)]	Cytosorb^®^ + Toraymixin^®^	6 (46.2)
**Low IgG** [n (%)]		9 (69.2)
**Cytokine HA duration** [hours, m (SD)]		50 (34)
**Length of ICU stay** [days, m (SD)]		33 (40)
**Length of hospital stay** [days, m (SD)]		54.85 (42)
**ICU mortality** [n (%)]		3 (23.1%)
**In hospital mortality** [n (%)]		3 (23.1%)

APACHE—Acute Physiology And Chronic Health Evaluation; BAL—Bronchoalveolar lavage; CRRT—continuous renal replacement therapy; ECMO—extracorporeal membrane oxygenation (VA—venoarterial, VV—venovenous); FLUB-V—Influenza-B virus; HA—hemoadsorption; ICU—intensive care unit; IMV—invasive mechanical ventilation; m—mean; n—number of population; PBC—peripheral blood culture; PID—Pelvic Inflammatory Disease; SD—standard deviation; SOFA—sequential organ failure assessment; SSTIs—Skin and Soft Tissue Infections.

**Table 2:** Analytical characteristics of the study population. The data have been expressed as “n” (%) if they are categorical and as median (interquartile range) or mean (standard deviation) if they are quantitative.

Analytical Characteristics of the Study Population	
**Leukocyte count** [(/mm^3^) m (SD)]	6670 (6868)
**Lymphocyte count** [(/mm^3^) m (SD)]	261 (180)
**Neutrophil count** [(/mm^3^) m (SD)]	5863 (6546)
**CRP** [(mg/dL) m (SD)]	25.83 (11)
**PCT** [(ng/mL) m (SD)]	167.33 (203)
**IL-6** [(pg/mL) m (SD)]	108,110.45 (104,325)
**IgG** [(mg/dL) m (SD)]	402 (230)
**IgM** [(mg/dL) m (SD)]	61.5 (29)
**IgA** [(mg/dL) m (SD)]	130 (77)
**EAA** [m (SD)]	0.93 (0.33)
**Platelet count** [(×10^9^/L) m (SD)]	105,000 (61,000)
**Ferritin** [(ng/mL) m (SD)]	2495 (3155)

CRP—C-reactive protein; EAA—Endotoxin Activity Assay; Ig—immunoglobulin, IL—interleukin; m—mean; n—number of population; PCT—procalcitonin; SD—standard deviation;.

**Table 3:** Distinguishing between phenotypes in patients with severe septic shock due, based on identifiable treatable traits.

Precision Medicine Strategies		Target (s)	Clinical Application
**Hyperinflammatory profile**	High endotoxinemia	Endotoxinemia	Rescue therapy Hemoadsorption
	Severe hypercytokinemia	Cytokine levels	
	Sequential hemoadsorption	Endotoxin and cytokine hemoadsorption	
**Low perfusion phenotype**	Patients with septic cardiomyopathy		ECMO venoarterial
**Low immunoglobulin phenotype**	Sepsis-associated hypogammaglobulinemia250 mg/kg/d by a 10 h infusion for 3 consecutive days		

## Data Availability

The datasets used and analyzed during the current study are available from the corresponding author upon reasonable request. The data are not publicly available due to preserve as much as possible the identity of the patients described.
